# A transcriptomic analysis of gene expression in the venom gland of the snake *Bothrops alternatus *(urutu)

**DOI:** 10.1186/1471-2164-11-605

**Published:** 2010-10-26

**Authors:** Kiara C Cardoso, Márcio J Da Silva, Gustavo GL Costa, Tatiana T Torres, Luiz Eduardo V Del Bem, Ramon O Vidal, Marcelo Menossi, Stephen Hyslop

**Affiliations:** 1 Departamento de Farmacologia, Faculdade de Ciências Médicas, Universidade Estadual de Campinas (UNICAMP), CP 6111, 13083-970, Campinas, SP, Brazil; 2 Centro de Biologia Molecular e Genética (CBMEG), Universidade Estadual de Campinas (UNICAMP), 13083-970, Campinas, SP, Brazil; 3 Departamento de Genética, Evolução e Bioagentes, Instituto de Biologia, Universidade Estadual de Campinas (UNICAMP), CP 6109, 13083-970, Campinas, SP, Brazil

## Abstract

**Background:**

The genus *Bothrops *is widespread throughout Central and South America and is the principal cause of snakebite in these regions. Transcriptomic and proteomic studies have examined the venom composition of several species in this genus, but many others remain to be studied. In this work, we used a transcriptomic approach to examine the venom gland genes of *Bothrops alternatus*, a clinically important species found in southeastern and southern Brazil, Uruguay, northern Argentina and eastern Paraguay.

**Results:**

A cDNA library of 5,350 expressed sequence tags (ESTs) was produced and assembled into 838 contigs and 4512 singletons. BLAST searches of relevant databases showed 30% hits and 70% no-hits, with toxin-related transcripts accounting for 23% and 78% of the total transcripts and hits, respectively. Gene ontology analysis identified non-toxin genes related to general metabolism, transcription and translation, processing and sorting, (polypeptide) degradation, structural functions and cell regulation. The major groups of toxin transcripts identified were metalloproteinases (81%), bradykinin-potentiating peptides/C-type natriuretic peptides (8.8%), phospholipases A_2 _(5.6%), serine proteinases (1.9%) and C-type lectins (1.5%). Metalloproteinases were almost exclusively type PIII proteins, with few type PII and no type PI proteins. Phospholipases A_2 _were essentially acidic; no basic PLA_2 _were detected. Minor toxin transcripts were related to L-amino acid oxidase, cysteine-rich secretory proteins, dipeptidylpeptidase IV, hyaluronidase, three-finger toxins and ohanin. Two non-toxic proteins, thioredoxin and double-specificity phosphatase Dusp6, showed high sequence identity to similar proteins from other snakes. In addition to the above features, single-nucleotide polymorphisms, microsatellites, transposable elements and inverted repeats that could contribute to toxin diversity were observed.

**Conclusions:**

*Bothrops alternatus *venom gland contains the major toxin classes described for other *Bothrops *venoms based on trancriptomic and proteomic studies. The predominance of type PIII metalloproteinases agrees with the well-known hemorrhagic activity of this venom, whereas the lower content of serine proteases and C-type lectins could contribute to less marked coagulopathy following envenoming by this species. The lack of basic PLA_2 _agrees with the lower myotoxicity of this venom compared to other *Bothrops *species with these toxins. Together, these results contribute to our understanding of the physiopathology of envenoming by this species.

## Background

Studies of the global composition of snake venoms have advanced dramatically in recent years with the introduction of transcriptomic and proteomic approaches for analyzing venom gland gene expression and venom composition [1-3]. The introduction of these two major fields in toxinology has created new perspectives for identifying novel molecules for drug discovery [4,5] and for improving antivenom development and the clinical treatment of snakebite [6-8]. A combination of these two approaches can be particularly useful in providing a more comprehensive understanding of venom composition [3,9-12].

Detailed transcriptomic studies of snake venom glands began with the report of Junqueira-de-Azevedo and Ho [13] who analyzed expressed sequence tags (ESTs) from the venom gland of the Brazilian pitviper *Bothrops insularis*, a species endemic to the island of Queimada Grande off the coast of the State of São Paulo in southeastern Brazil. Since then, venom gland transcriptomes have been reported for various other South American venomous snakes, including *Bothrops *species [11,14-16], *Crotalus durissus collilineatus *(South American rattlesnake) [17], *Lachesis muta *(bushmaster) [18], *Micrurus corallinus *(coral snake) [19] and the colubrid *Philodryas olfersii *[20].

The genus *Bothrops *is responsible for most cases of snakebite throughout Latin America [21] and in Brazil accounts for 80-90% of bites by venomous snakes. Envenoming by *Bothrops *species results in extensive local effects, including pain, edema, inflammation, hemorrhage and necrosis [22-24], as well as systemic actions that include coagulopathy, internal hemorrhage, circulatory shock and acute renal failure [21,25-28]. These actions are mediated by a variety of venom components, with the most extensively studied being (hemorrhagic) metalloproteinases [29] and myotoxic phospholipases A_2 _(PLA_2_) [23,30,31].

*Bothrops alternatus *occurs in southeastern and southern Brazil, Uruguay, central and northern Argentina and south-eastern and southern Paraguay [32]. This species is an important cause of snakebite, although the prevalence of bites varies considerably throughout its geographic distribution depending on the regional human and snake population densities and the presence of more abundant species such as *Bothrops jararaca*, the major cause of snakebite in southeastern Brazil. Thus, for example, notifications of bites by Brazilian *Bothrops *spp. received by the Instituto Butantan from 1902-1945 (n = 4,881) suggest that *B. alternatus *accounts for ~6% of bites by this genus [33]. In contrast, in a more recent series of 3,139 cases attended at the Hospital Vital Brazil, Instituto Butantan in São Paulo, from 1981-1990, only two (0.14%) of the 1,412 cases in which the *Bothrops *species was identified were caused by *B. alternatus *compared to 1,376 (97.5%) by *B. jararaca *[34]. In regions where *B. alternatus *is more abundant or predominates (southern Brazil, Uruguay and parts of Argentina), this species contributes to much a greater proportion of snakebites, e.g., 18% in Argentina, but may be much higher locally (≥ 60% of snakebites in the Argentinian province of Corrientes) [35].

*Bothrops alternatus *produces clinical manifestations characteristic of this genus [21,28]. In a series of 32 cases involving this species [36], most of the patients (74.2%) were 15-49 years old and were bitten in the lower limbs (84.3%). All patients developed local pain, edema and most (31; 96.9%) had incoagulable blood, 13 (40.6%) developed hemorrhage, 7 (21.9%) had blisters and 3 (9.4%) showed necrosis; there were no fatalities. In addition, renal failure has been observed after bites by this species in Uruguay [37]. The administration of antivenom (produced by the Instituto Nacional de Producción de Biológicos - INPB in Argentina and the Instituto Butantan, Fundação Ezequiel Dias or Instituto Vital Brazil in Brazil) is the standard treatment for envenoming by *B. alternatus*. These antivenoms may be bivalent (raised against *B. alternatus *and *B. neuwiedii *in Argentina) or poplyvalent (raised against the clinically most relevant *Bothrops *species in Argentina and Brazil - *B. alternatus*, *B. jararaca*, *B. jararacussu*, *B. neuwiedi *and *B. moojeni*) [38,39]. In the case series studied by Bauab et al. [36], a median of 40 ml of antivenom (four 10 ml ampoules) was administered.

Although *B. alternatus *venom contains a variety of enzymatic and biological activities, relatively few of the venom proteins involved have been purified and characterized, with most investigations having dealt with metalloproteinases and disintegrins [40-50], PLA_2 _[51-55], coagulant enzymes [56-58], L-amino acid oxidase (LAO) [59] and phosphodiesterase [60]. Despite these investigations, the venom of *B. alternatus *is still less understood than those of other *Bothrops *species such as *B. jararaca *and *B. jararacussu*. In order to obtain a more comprehensive understanding of the toxinology of this species, we have used a transcriptomic approach to examine venom gland gene expression in *B. alternatus *and compared the results with those for other members of this genus.

## Methods

### Venom glands and RNA isolation

Venom glands from three adult *B. alternatus *snakes were obtained three days after venom extraction. The choice of this interval was based essentially on other studies [16,17,61,62] that used this period, although intervals of two [14] and four [11,13] days post-milking have also been used for *Bothrops *species. The choice of a 2-4 day post-milking interval prior to gland removal is partly based on studies of venom mRNA and protein synthesis in snake venom glands that show maximal production 3-8 days post-milking [reviewed in [63]], and partly on histological and functional studies in *B. jararacussu *[64] and *B. jararaca *[65], respectively, showing that changes in gland epithelial morphology and venom production peak around four days post-milking.

Each pair of venom glands was homogenized in liquid nitrogen and total RNA was extracted with TRIzol reagent (Invitrogen, UK), according to the manufacturer's instructions. Three cDNA libraries (one from each snake) were independently constructed with CloneMiner cDNA library construction kits (Invitrogen, UK), according to the manufacturer's instructions. The cDNA libraries were processed and analyzed using an in-house bioinformatics pipeline, with all annotations being done manually (contig by contig and singlet by singlet) for each library. The production of three independent libraries meant that it was possible to undertake detailed analyses of SNPs, microsatellites and inversions not generally done for *Bothrops *species since in most studies the venom glands of different snakes are usually combined into a single pool for mRNA extraction, with a subsequent loss of information.

This work was approved by the institutional Committee for Ethics in Animal Experimentation (CEEA/UNICAMP, protocol no. 864-1) and was done according to the ethical guidelines of the Brazilian Society of Laboratory Animal Science (SBCAL, formerly the Brazilian College for Animal Experimentation - COBEA).

### Sequencing

The cDNA libraries were sequenced using BigDye terminator 3.0 kits and an automated DNA capillary sequencer (ABI PRISM 3700 DNA Analyzer, Applied Biosystems, Foster City, CA, USA). All of the cDNA sequences were 5' sequenced using the primer M13F (5'-TGTAAAACGACGGCCAGT-3').

### Clusterization, assembly and identification of *Bothrops alternatus *expressed-sequence tags

The Phred program [66] was used to obtain sequences and quality files from chromatograms obtained from expressed-sequence tag (EST) sequencing. The EST cleaning pipeline described by Baudet and Dias [67] was then used to pre-process the ESTs and prepare the sequences for assembly. This pipeline removes sequences with plasmid similarity, polyA/polyT regions, low base quality and slippage signals. Sequences <100 bp long after cleaning were discarded. CAP3 software [68] was used to cluster and assemble the clean sequences into contigs and singlets (unisequences). For assembly, an overlap of 100 bp and an identity of at least 95% were used as criteria to detect pairwise similarities.

### Annotation of *Bothrops alternatus *ESTs

After clustering and assembly, a BLAST search was done to identify similarities between the ESTs and sequences deposited in public databases. All of the sequences were aligned against the GenBank non-redundant (nr) protein database using BLASTX and BLASTN [69] with an E-value cut-off of 1e-5. The *B. alternatus *ESTs were also screened against two locally generated sequence databases, SerpP and SerpN, that included all snake protein and nucleotide sequences from GenBank, respectively. In addition, the ESTs were compared with the complete genome of the lizard *Anolis carolinensis *(http://genome.ucsc.edu/cgi-bin/hgGateway?db=anoCar1). Gene Ontology annotation was done with Blast2GO [70] using GO-slim terms. The uncharacterized ESTs were examined for the presence of a signal peptide by using SignalP 3.0 software (http://www.cbs.dtu.dk/services/SignalP/).

### Sequence alignments

Sequence alignments for selected proteins were done with the program ClustalW [71].

### Single nucleotide polymorphisms

The software QualitySNP [72] was used to identify single-nucleotide polymorphisms (SNPs). Non-synonymous and synonymous SNPs (nsSNPs and sSNPs, respectively) were identified by detecting open-reading frames (ORFs) of contigs with SNPs using the FASTA algorithm run against the 2008 version of UniProt [73]. The possibility of SNPs arising from artifacts during DNA sequencing was minimized by the fact that the cDNA libraries were prepared independently from three snakes and that we used consensus sequences from contigs with at least three reads from separate sequencing plates for which the cDNA was prepared and the reactions run on different days. These procedures considerably reduced the possiblity of artifacts derived from DNA sequencing and strengthened our conclusions regarding the presence of SNPs.

### Identification of transposable elements and long inverted repeats

Alignment of the unisequences to repetitive elements in RepBase release 14.08 [74] was done with BLASTN that was automated using in-house Perl scripts (available upon request). The E-value cut-off was set at 1 × 10^-10 ^and only alignments of at least 50 bp were considered for unisequences. In addition, the alignments with database sequences had to show >80% identity over at least 10% of their lengths. To identify possible functional integrations of transposable elements (TEs) into host genes, we searched for chimeric transcripts between TEs and protein-coding genes. Unisequences that aligned with a TE in the database over 10% to 90% of their sequence were used to match the sequence back to the corresponding host gene by masking the element in the sequence and querying the NCBI nr nucleotide database. For tBLASTX remote searches, a minimum of 50% of the sequence was required to be involved in the best hit, with at least 70% identity.

Additional repeated elements that were not present in RepBase were observed in some unisequences. These novel elements were identified using the palindrome program in the EMBOSS package [75]. Unisequences that contained inverted repeats were identified by aligning each transcript against itself using BLASTN (E-value cut-off of 1e-5) and then inspected visually to check for alignments in opposite strands. The upper limit for mismatches between the two repeated segments was 10%.

### Comparison of the *B. alternatus* EST library with other *Bothrops* species

The pattern of gene expression in *B. alternatus *venom gland was compared with EST data for other *Bothrops *species (*B. atrox *[16], *B. insularis *[11,13], *B. jararaca *[15] and *B. jararacussu *[14]) available in public databases, and with the major toxin classes detected by proteomic analyses of *Bothrops *venoms. In this comparison and in the description of the different toxin groups discussed below, the relative percentages of the venom gland EST categories were calculated as a percentage of the overall (total) number of all ESTs identified, whereas the relative percentages of the ESTs related to specific toxin groups was calculated based on the total number of toxin ESTs identified.

## Results and Discussion

### Venom gland EST database

**Figure 1:**
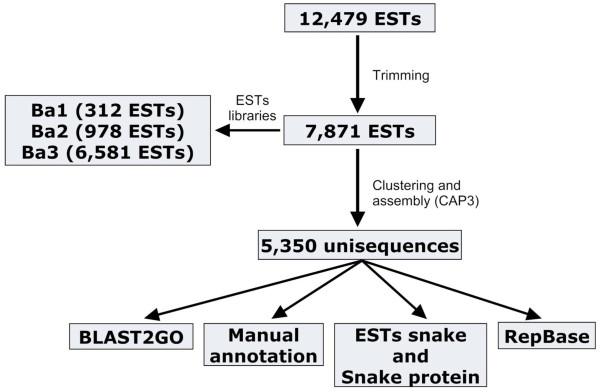
**Bioinformatics pipeline for clustering, assembling and annotation of *B. alternatus *ESTs**. A total of 12,479 unisequences were generated from three libraries (*B. alternatus *libraries Ba1, Ba2 and Ba3). After trimming, 7,871 ESTs yielded 5,350 valid unisequences.

**Figure 2:**
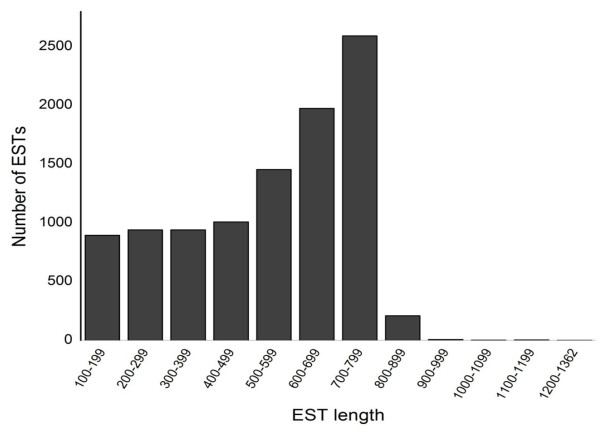
**Size distribution of *B. alternatus *venom gland ESTs.** Most of the 5,350 ESTs were 500-800 bp in length.

**Figure 3:**
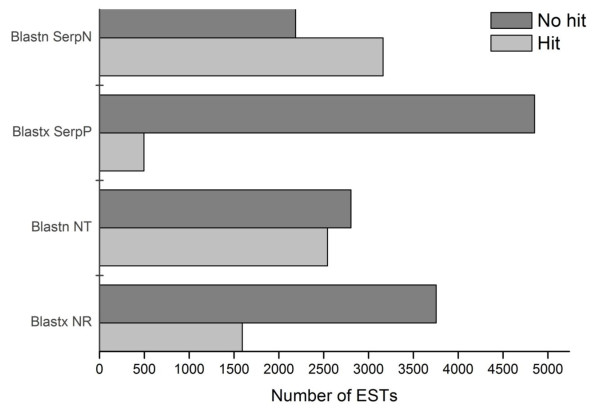
**Comparison of the number of hits and no-hits in BLAST searches of selected databases**. NT and NR: GenBank nucleotide and non-redundant databases, respectively. SerpP and SerpN: snake protein and nucleotide databases, respectively.

cDNA libraries were generated from three pairs of *B. alternatus *venom glands and analyzed using the pipeline shown in Figure 1. A total of 5,350 valid unisequences was obtained by single-pass sequencing and, after trimming, the lengths of the sequences ranged from 100 bp to 848 bp (mean: 562 bp) (Figure 2). The ESTs were assembled into 838 contigs and 4512 singletons. Thirty percent of the sequences (1,605 ESTs) were hits, of which 1,245 (23% of total ESTs and ~78% of hits) were related to toxins; the remaining 70% (3,745 ESTs) were no-hits. Figure 3 shows the proportion of hits and no-hits for selected databases. The proportion of hits was greater against the GenBank and SerpN nucleotide databases compared to the corresponding protein databases. There was a particularly low number of hits in SerpP, which likely reflects the limited number of non-venom snake protein sequences available in public databases.

SignalP analysis identified 147 ESTs with signal peptides, 62 of which were related to snake toxins (37 for PLA_2_, 7 for metalloproteinases, 6 for proteins related to platelet glycoprotein 1β binding protein, 6 for proteins related to ACF 1/2 β-chain, 4 for serine proteinases, and 1 each for disintegrins and bradykinin-potentiating peptides or BPPs), 50 were for other proteins and 35 were for unknown proteins.

All of the ESTs were deposited in the dbEST division of GenBank (accession numbers GW575430 to GW583300).

### Gene ontology (GO)

**Figure 4:**
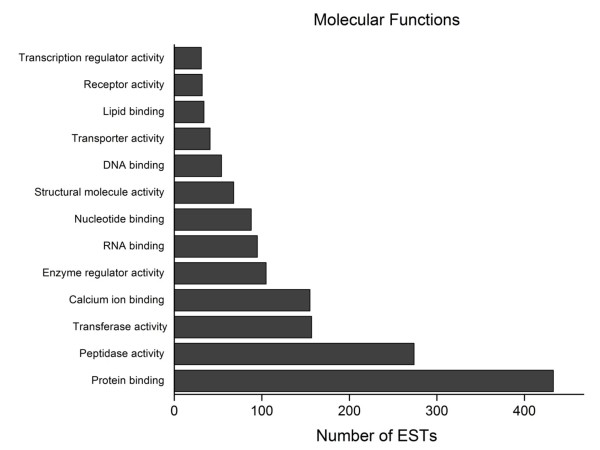
**Molecular functions identified by Gene Ontology (GO) analysis of *B. alternatus *venom gland ESTs**. The X-axis shows the number of ESTs and the Y-axis the GO terms.

**Figure 5:**
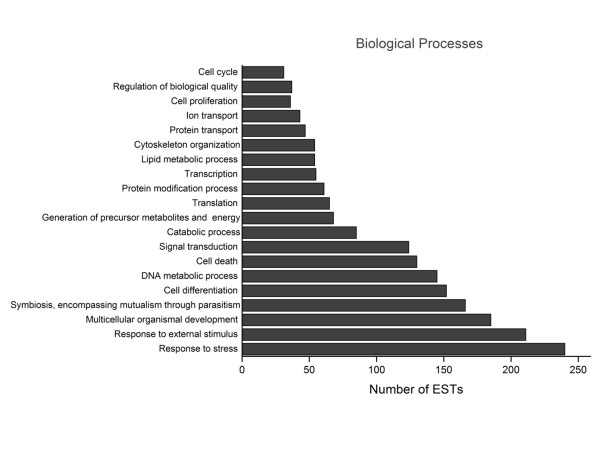
**Biological processes identified by Gene Ontology (GO) analysis of *B. alternatus *venom gland ESTs**. The X-axis shows the number of ESTs and the Y-axis the GO terms.

A global GO analysis [70] of unisequences in relation to molecular function revealed that the largest number of transcripts was related to protein binding, followed by peptidase and transferase activities, calcium-binding, enzyme regulation, nucleic acid binding, structural protein and transporter activities, lipid binding, and transcriptional regulation (Figure 4). Analysis of the biological processes revealed two major groups arbitrarily defined as those with >100 ESTs and those with ≤100 ESTs. The former contained genes related to stress, responses to external stimuli, development, symbiosis, cell differentiation, DNA metabolism, cell death and signal transduction, whereas the latter consisted of genes related to catabolism, precursor biosynthesis, translation, protein modification, transcription, lipid metabolism, protein and ion transport, cytoskeletal organization, cell proliferation and cell cycle (Figure 5). The biological processes identified here generally agreed with the broad categories (general metabolism, transcription and translation, processing and sorting, (polypeptide) degradation, structural functions and cell regulation) reported for other *Bothrops *species [11,13-16] and *L. muta *[18].

### Selected venom gland proteins

#### Venom gland receptors

We detected one transcript coding for a protein similar to α_1D_-adrenoceptor from *B. jararaca*. Since α- and β-adrenergic mechanisms have an important role in venom production in *B. jararaca *[76-78], principally through receptors structurally related to α_1D_- and α_1B_-adrenoceptors [78], a similar regulatory mechanism mediated by these receptors probably also occurs in *B. alternatus*. Another receptor identified was vascular endothelin type A (ET_A_) receptor (one singlet). This receptor mediates the vasoconstrictor activity of the endothelin family of peptides in *B. jararaca *aorta [79] and could be important in the regulation of vascular tone in blood vessels irrigating the venom gland.

A contig was identified for a calglandulin-like EF-hand protein. Venom gland calglandulin, initially identified in *B. insularis *based on an EST analysis of this venom gland [13] and subsequently cloned and expressed from this species [80] (also detected in elapid venom glands [9,81]), is a Ca^2+^-binding protein structurally similar to calmodulin and troponin C. This protein, which is venom gland-specific (not found in other snake organs or venom) [80], may have a role in the transport and secretion of venom toxins, in addition to possibly acting as an intracellular Ca^2+^-chelator to regulate toxin activity.

#### Dusp6

**Figure 6:**
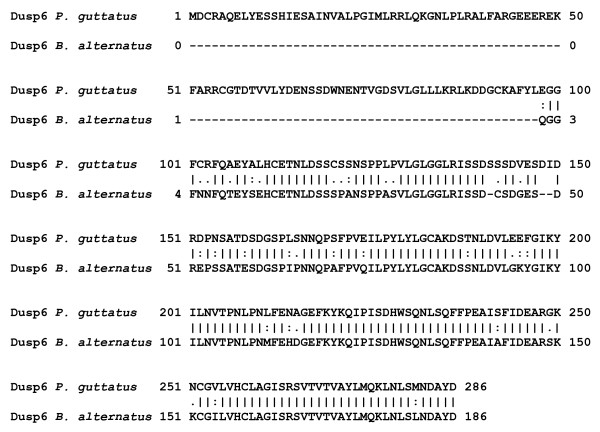
**Amino acid sequence alignment of *B. alternatus *Dusp6 with that of *Pantherophis guttatus *(corn snake)**. The proteins showed greater similarity in the second half of their sequences. Insertions or deletions are represented by gaps (-), vertical bars (|) indicate identical residues, two dots (:) indicate strongly similar residues and one dot (.) indicates weakly similar residues.

Dual specificity phosphatases (Dusp) have important functions in embryogenesis, cell growth and immune responses by acting as negative feedback regulators of mitogen-activated protein kinases (MAPK) [82]. *DUSP6 *has an important role in snake embryogenesis, with high gene expression in the anterior part of the presomitic mesoderm [83,84]. This protein has not previously been detected in venom gland transcriptomes or in analyses of venom composition. We detected a partial sequence for *DUSP6 *protein that shared similarity with the corresponding protein from *Pantherophis guttatus *(corn snake; accession number ABW82165) (Figure 6). The role of *DUSP6 *in the venom gland is unclear but may be related to development of the gland secretory epithelium and venom production.

#### Thioredoxin

**Figure 7:**
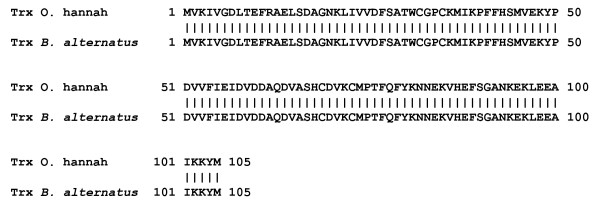
**Amino acid sequence alignment of thioredoxin (Trx) from *B. alternatus *and *O. hannah***. The sequences deduced from venom gland cDNA were identical.

Thioredoxin (Trx) is a protein with a variety of activities, including roles in DNA synthesis, protein disulfide bond reduction and the degradation of H_2_O_2 _that may be related to protection against oxidative stress and the induction of apoptosis [85]. Thioredoxin participates in redox reactions through the reversible oxidation of its active center dithiol to a disulfide, thereby catalyzing dithiol-disulfide exchange reactions; the reduction of the active site disulfide of oxidized Trx to regenerate the dithiol of reduced Trx is catalyzed by thioredoxin reductase, a selenium-containing flavoprotein [86]. Trx is also involved in the reversible S-nitrosylation of cysteine residues in target proteins, an important step in signaling by intracellular nitric oxide (NO). We found a protein sequence in the *B. alternatus *transcriptome that was related to Trx previously identified in venom gland cDNA from *O. hannah *(accession number AAK09384) (Figure 7). The function of Trx in venom glands is unknown, but it may be involved in protecting epithelial secretory cells of the gland from oxidative stress and death (by necrosis or apoptosis), particularly since venom components such as LAO can cause cell death through the formation of H_2_O_2 _[87,88].

### Venom components identified by ESTs

**Figure 8:**
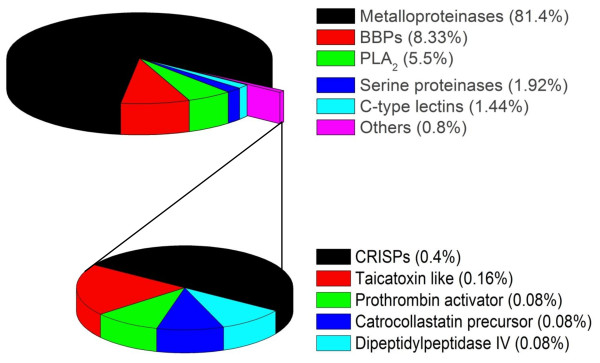
**Principal toxin classes in the *B. alternatus *venom gland EST library**. The upper pie chart shows the major toxin classes detected and the lower pie chart shows the minor components (part of the "Others" group in the upper panel). The percentages indicate the abundance of each class relative to the total number of toxin-related ESTs in the library. BLAST only against nr hits with protein.

Figure 8 summarizes the venom components detected based on EST analysis. The major classes detected were metalloproteinases/disintegrins, BPP/C-type natriuretic peptide (CNP) precursors, PLA_2_, serine proteinases and C-type lectins. Genes expressed in lower abundance included cysteine-rich secretory proteins (CRISPs), taicatoxin-like protein, prothrombin activator, a catrocollastatin precursor and dipeptidylpeptidase IV (DPP IV).

### Major toxin classes

#### Metalloproteinases/disintegrins

**Figure 9:**
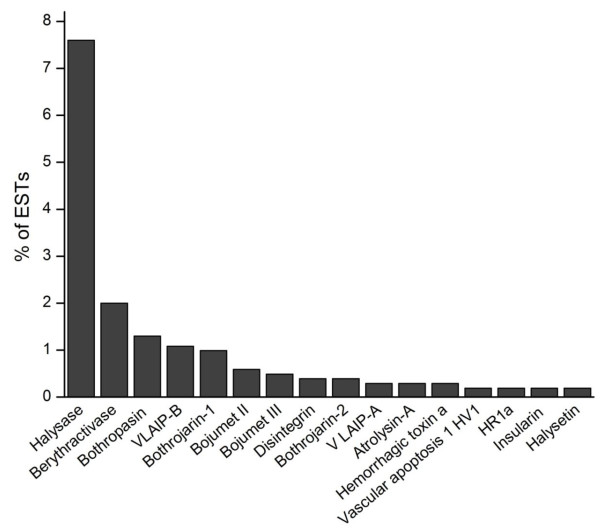
**Distribution of metalloproteinase transcripts in the *B. alternatus *venom gland library**. The frequency (%) of transcripts similar to other known metalloproteinases is shown. Transcripts with very low frequencies (< 0.2%; not shown) included those similar to vascular apoptosis-inducing protein 2A, jararhagin precursor, contortrostatin and halystatin. BLAST only against nr hits with protein.

*Bothrops *venoms are rich in a variety of snake venom metalloproteinases (SVMPs) that contribute to local and systemic bleeding after envenoming [21,28,29]. SVMPs are currently classified in three major classes (PI, PII and PIII, with the latter two containing five and four subclasses, respectively) [89], with PIII metalloproteinases being particularly abundant and extensively studied in *Bothrops *venoms. Metalloproteinases were the most abundant venom components in *B. alternatus *venom gland (Figure 8) and were almost exclusively class PIII proteins. SVMPs accounted for 1010 ESTs that were grouped into 22 contigs, the most abundant of which were related to jararhagin from *B. jararaca*, Russell's viper (*Vipera russelli*) venom factor × heavy chain (RVV-X) and hemorrhagic factor 3 (HF3), also from *B. jararaca *(all class PIII proteins) (Figure 9). Hits were also obtained for *Bothrops *metalloproteinases such as berythactivase from *B. erythromelas*, bothropasin from *B. jararaca *and metalloproteinases II (Bojumet II) and III (Bojumet III) from *B. jararacussu*. There was a low number of transcripts for class PII proteins, including one transcript coding for contortrostatin and one contig related to insularinase. No class PI SVMPs were detected. The overwhelming abundance of transcripts for class PIII SVMPs (with few PII and no PI SVMPs) observed here agrees well with the results of a proteomic analysis of this venom in which only class PIII SVMPs were detected [90]. Our finding of transcripts related to jararhagin and berythactivase also agrees with these authors who detected proteins related to these two SVMPs in *B. alternatus *venom.

In agreement with the abundance of SVMP transcripts, two PIII metalloproteinases have been isolated and characterized from this venom. Souza *et al. *[40] reported the characterization of a 55 kDa PIII metalloproteinase (alternagin) from Brazilian *B. alternatus *venom that can undergo autolysis to release a 28 kDa ECD-disintegrin-like cysteine-rich domain (alternagin-C). Alternagin inhibits the binding of K562 cells to collagen by selectively blocking α_2_β_1 _integrin, in a manner similar to jararhagin from *B. jararaca *venom; this inhibitory action is apparently mediated by alternagin-C. Subsequently, Gay *et al. *[44,45] also isolated alternagin (which they termed balteragin) from Argentine *B. alternatus*. This protein causes edema, hemorrhage and necrosis when administered intramuscularly in mice, and systemic hemorrhage, primarily in the lungs, kidneys and liver, when administered intravenously. Cominetti *et al. *[42] described the characterization of a 130 kDa dimeric PIII metalloproteinase/RDG-disintegrin (BaG; monomeric form = 55 kDa) from Brazilian *B. alternatus *venom. This enzyme, which accounts for at least 0.2% of venom protein, inhibits ADP-induced platelet aggregation via a mechanism independent of its enzymatic activity. BaG also blocks the adhesion of K562 cells to fibronectin (and detaches cells previously adhered to this protein), a phenomenon mediated by binding to α_5_β_1 _integrin, but has no effect on the adhesion of these cells to collagen type I (mediated by α_2_β_1 _integrin).

The processing of PII and PIII SVMPs gives rise to RDG-disintegrins and ECD-disintegrin-like cysteine-rich (DC) domains, respectively, that exert a variety of biological activities through interaction with cell surface integrins [89]. In the *B. alternatus *ESTs, there were four ESTs coding for disintegrins, and at least three disintegrins were identified in a proteomic analysis of this venom [90]. As indicated above, autolysis of alternagin gives rise to alternagin-C, an ECD-disintegrin-like cysteine-rich domain [40]. Alternagin-C exerts a variety of activities, including the induction of neutrophil migration via integrin signaling [41], stimulation of human umbilical vein endothelial cell migration [43,46] and modulation of angiogenesis *in vitro *and *in vivo *[47], but has a limited beneficial effect on muscle regeneration [48]. More recently, another disintegrin (DisBa-01) identified in a cDNA library from *B. alternatus *venom gland, has been shown to inhibit angiogenesis and melanoma mestastasis by interacting with α_v_β_3 _integrin [49] and prevents platelet adhesion to fibrinogen, thereby prolonging bleeding time in mice [50]; the latter action is mediated by interaction with platelet α_IIb_β_3 _integrin.

#### Phospholipase A_2_

*Bothrops *venoms are rich in acidic and basic PLA_2 _with Asp or Lys at position 49 in their active site. Many of these PLA_2 _cause pain, edema, inflammation and myotoxicity [23,30], as well as neuromuscular blockade *in vitro *[91]. The content of myotoxic PLA_2 _varies considerably among *Bothrops *species, e.g., *B. asper, B. colombiensis, B. fonsecai, B. jararacussu*, *B. moojeni*, *B. neuwiedi *and *B. pradoi *have an elevated content of these enzymes whereas others, such as *B. alternatus*, *B. atrox, B. cotiara, B. erythromelas *and *B. jararaca*, have few or no myotoxins [6,92-96]. In addition, some species, e.g., *B. jararacussu *[14,97] contain a variety of PLA_2 _in addition to myotoxins. PLA_2 _accounted for 5.6% of *B. alternatus *venom components (1.28% of the total EST library) (Figure 8). This PLA_2 _content was similar to that of *B. insularis *(6.7%) [13] and *B. jararaca *(6.7%) [15], but considerably less than for *B. atrox *(14.6%; [16]) and *B. jararacussu *(58%; [14]). The *B. alternatus *PLA_2 _formed two major groups: those related to BOTIN PLA_2 _(PA2-BOTIN or BinTX-I; SwissProt/trEMBL accession number Q8QG87), one of two acidic Asp49 PLA_2 _isoforms (BinTX-I = pI 5.05; BinTX-II = pI 4.49) characterized from the venom of *B. insularis *[98], with 41 ESTs, and those related to an acidic (pI 4.5) hypotensive Asp49 PLA_2 _(Q8AXY1) from *B. jararacussu *venom [99], with 28 ESTs.

BinTX-I causes mouse hind-paw edema, as well as myonecrosis and partial neuromuscular blockade in chick biventer cervicis preparations [98]. Hence, it is probable that the corresponding PLA_2 _in *B. alternatus *venom exert similar effects. In contrast, the acidic hypotensive PLA_2 _from *B. jararacussu *is not myotoxic, cytotoxic or lethal but causes edema and hypotension, and inhibits platelet aggregation [99]. Nisenbom *et al. *[51,52] reported the characterization of an acidic (pI 5.04-5.08) PLA_2 _from *B. alternatus *that accounted for most of the lethality of this venom in mice and produced cardiovascular alterations such as dyspnea, tachycardia, arrhythmia and circulatory shock, as well as tissue damage (hemorrhage, necrosis, etc.). This enzyme therefore shares properties with *B. insularis *and *B. jararacussu *PLA_2_.

Basic Lys49 PLA_2_s characteristic of *Bothrops *venoms were not detected in this venom gland library, in agreement with a proteomic analysis of this venom [90] and with EST analyses of *B. insularis *[13] and the bushmaster *L. muta *[18]. However, two ESTs coding for the basic PLA_2 _of crotoxin (the principal neurotoxin of *C. d. terrificus*; also identified in *B. insularis *ESTs; [13]) and one transcript for the PLA_2 _β-neurotoxin ammodytoxin (from *Vipera ammodytes*) were detected. The general absence of basic Lys49 PLA_2 _in the *B. alternatus *transcriptome agrees with the very low content of myotoxic PLA_2 _already reported for this venom [93,95]. Indeed, to date, only one basic (pI 8.63) myotoxic Lys49 PLA_2 _has been isolated and characterized from this venom [54,55]. This PLA_2_, which accounts for <1% of the venom content, causes mouse footpad edema, skeletal muscle myonecrosis, lysis of C2C12 skeletal muscle myoblasts and presynaptic neuromuscular blockade in chick biventer cervicis and mouse phrenic nerve-diaphragm preparations. However, given the very low level of this PLA_2 _in the venom, its overall contribution to the activities of the venom is likely to be minimal.

In addition to PLA_2_, inhibitors of this enzyme were also detected (17 ESTs with hits only in the 3'UTR; similar to PLA_2 _inhibitors of *Trimeresurus flavoviridis*). Venom gland PLA_2 _inhibitors show considerable sequence identity with γ-PLI (phospholipase inhibitors) identified in snake sera, including that of *B. alternatus *[100].

#### Serine proteinases

Coagulopathy is a major complication of systemic envenoming by *Bothrops *species that, in conjunction with the action of venom metalloproteinases, can contribute to systemic hemorrhage [21,28]. Coagulation disturbances caused by *Bothrops *venoms are mediated by a variety of enzymes that act at various points of the coagulation cascade, including fibrinogen degradation and fibrin consumption. The clinical outcome of this combined action is incoagulable blood. Some of the serine proteinases may also have kinin-releasing activity that can contribute to local pain, vasodilation and systemic hypotension [101,102]. In the *B. alternatus *transcriptome, serine proteinases were the fourth most abundant group of toxins (Figure 8) (1.9% and 0.45% of toxin and total transcripts, respectively), with ~42% (10 ESTs) being related to BthaTL, a serine proteinase identified in this venom by Vitorino-Cardoso *et al. *[58] based on gene cloning and amino acid sequence analysis. This protein, which shares sequence identity (> 60%) with a variety of snake venom thrombin-like enzymes (TLE), is probably the same as the TLE balterobin previously purified and characterized by Smolka *et al. *[57]. Other proteins in this group were related to serine proteinase isoform 5 (7 ESTs), venom serum proteinase-like protein 2 precursor (5 ESTs) and venom serine proteinase HS112 precursor (1 EST) and TLE (1 EST). In addition, there was one transcript with a hit in a UTR for a protein similar to KN-BJ2, a kinin-releasing, fibrinogen clotting enzyme from *B. jararaca *venom [15,102,103]. The range of serine proteinases identified here in ESTs agrees with the presence of coagulant (BthaTL and several TLEs homologous to ancrod, batroxobin, proteinase HS114 and pallase) and kinin-releasing enzymes identified in the proteome of this venom [90]. The abundance of serine proteinases in the *B. alternatus *transcriptome was similar to the 2% reported for *B. jararacussu *[14] but considerably less than in other species of this genus, e.g., ranging from 8.2% in *B. atrox *[16] to 28.6% in *B. jararaca *[15]. Differences in the relative proportion of serine proteinases present in the venoms could contribute to variations in the severity of coagulopathy among *Bothrops *species.

#### C-type lectins

Proteins with C-type lectin-like domains are widespread in the animal kingdom, including venoms. Venom C-type lectins form two major groups: true C-type lectins with carbohydrate binding domains and C-type lectin-like proteins with non-carbohydrate binding domains, the latter consisting mainly of factor IX/X binding proteins and proteins that interact with platelet receptors. C-type lectins are involved in venom-induced coagulation disturbances and have been identified in various *Bothrops *ESTs [11,13-16]. These proteins formed the fifth most abundant class of toxin transcripts in *B. alternatus *(18 ESTs with protein, 1.46%, or 0.33% of the total cDNA library) (Figure 8) and were related to the α subunit of platelet glycoprotein Ib-binding protein (8 ESTs), the ACF 1/2 β chain (7 ESTs), coagulation factor IX-binding protein (2 ESTs) and bothrojaracin (1 EST). There was also a transcript for convulxin (one singlet in the 3'UTR), a C-type lectin initially identified in the venom of *C. d. terrificus *(also identified in *B. insularis *ESTs; [13]). The abundance of C-type lectins was considerably less than in *B. atrox *(4.8%; [16]), *B. insularis *(14.5%; [13]), *B. jararaca *(8.3%; [15]) and *B. jararacussu *(5%; [14]); if these relative proportions are reflected in the venoms, then C-type lectins may contribute less to coagulopathy in bites by *B. alternatus *than by other *Bothrops *spp.

Few proteins of this class have been characterized from *B. alternatus *venom. Bothrojaracin, a 27 kDa heterodimer (13 kDa and 15 kDa) initially purified from *B. jararaca *venom, is homologous to several C-type lectin family proteins and inhibits thrombin activity, e.g., platelet aggregation and fibrinogen clotting, by interacting with exosites I and II of this enzyme to form a non-covalent complex [104,105]. Castro *et al. *[56] characterized bothroalternin, a bothrojaracin-like protein, from *B. alternatus *venom; this protein 27 kDa homodimer cross-reacts with antibodies to bothrojaracin, shares sequence identity (68-84%) with the α and β chains of factor IX/X binding protein and botrocetin from *B. jararaca *venom, and inhibits thrombin-induced platelet aggregation. Bothroalternin has also been detected in the proteome of *B. alternatus *[90].

Sugar-binding lectins, which are related to C-type lectins, have been detected in some *Bothrops *ESTs [11,14,16], but were not detected here or in *B. jararaca *[15].

#### Bradykinin-potentiating peptide and C-type natriuretic peptide precursors

*Bothrops *venoms have long been known to contain bradykinin-potentiating peptides (BPPs) that enhance venom-induced hypotension by inhibiting angiotensin-converting enzyme, a pivotal enzyme in the formation of angiotensin II (vasoconstrictor peptide) and degradation of bradykinin (vasodilator peptide) [106]. Numerous such peptides have since been identified in *Bothrops *venoms ([107] and references therein), and cloning and sequence analysis studies in the late 1990s identified genes encoding BPPs in *B. jararaca *venom gland [108,109]. These same studies also reported the presence of genes encoding for C-type natriuretic peptides (CNPs) that could contribute to venom-induced hypotension. Transcriptomic studies of *Bothrops *venom glands have confirmed these findings and shown that genes encoding these two peptides account for 6-20% of venom toxin ESTs [11,13,15,16]. A similar percentage (~9%) was observed here for *B. alternatus *(Figure 8), for which 110 ESTs coded for BPP/CNP precursors (specifically the CNP region) while another 156 ESTs showed similarities only in the 3'UTR. In contrast to the abundance of BPP/CNP transcripts detected in *Bothrops *spp. by EST analysis, proteomic analyses have shown that these peptides account for <1% of the venom protein/peptide content [6,110], although in *B. insularis *the venom BPP content is 10% [11]. A similar situation applies to CNPs for which some peptidomic analyses have detected no such peptides in venom, despite the identification of transcripts in EST analyses, e.g., *B. insularis *[11] and *L. muta *([111]; but cf. [10]) (see section on 'Inverted repeats' below for additional comment on CNP detection).

### Minor toxin classes

#### Cysteine-rich secretory proteins

Snake venom cysteine-rich secretory proteins (CRISPs) are 23-26 kDa proteins with a high content of cysteine residues (16) that form eight disulfide bridges. CRISPs are widespread in snake venoms and have been detected in transcriptomic [11,13-16] and proteomic [6,7,11,96,110,112,113] studies of *Bothrops *venoms, where they account for 0.5-2% and 0-3.6% of venom genes and toxins, respectively. In agreement with these studies, we detected five ESTs with protein (0.4% of toxin transcripts) for CRISPs in *B. alternatus *venom gland. Although the role of venom CRISPs remains poorly understood, several members of this family block a variety of ion channels, including L-type Ca^2+ ^channels, cyclic-nucleotide-gated ion channels, voltage-activated K^+ ^channels (Kv1.3), high-conductance Ca^2+^-activated K^+ ^channels (BCa) and the ryanodine-sensitive intracellular Ca^2+ ^channel. Peichoto *et al. *[114] have recently shown that a CRISP from the venom of the South American colubrid *Philodryas patagoniensis *causes myonecrosis. Since myonecrosis is an important local response to envenoming by *Bothrops *spp., including *B. alternatus*, it is possible that CRISPs could contribute to this activity.

#### Dipeptidylpeptidase IV

Dipeptidylpeptidase IV (DPP IV) (also known as CD26) has a wide distribution in snake venoms [115]. DPP IV genes have been identified in the venom glands of *B. jararaca *[15], *Gloydius blomhoffii brevicaudus *[116], *L. muta *[18] and various Australian elapids [9]. Gasparello-Clemente and Silveira [117] reported DPP IV in *Bothrops *venoms, with the highest activity in *B. alternatus*. In agreement with this, we detected a transcript coding for DDP IV protein in *B. alternatus*. Venom DPP IV, which has enzymatic properties similar to those of other eukaryotic DPP IV [116], may contribute to venom-induced cardiovascular alterations, possibly by degrading endogenous peptides through association with exosome-like vesicles in fresh venom [118], and possibly by interfering with glucose homeostasis and the immune and neuroendocrine systems [119]. DPP IV may also be important in processing polypeptide precursors of venom peptides, as suggested for wasp and bee venoms [120,121].

#### Growth factors

Snake venom growth factors comprise primarily vascular endothelial growth factor (svVEGF) and nerve growth factor (NGF), both of which have been detected in *Bothrops *ESTs [11,13-16]. We detected five ESTs for VEGF similar to *B. insularis *VEGF [13,122], one EST similar to *L. muta *VEGF [18] and one EST similar to *C. d. terrificus *NGF. However, the proportion of growth factor transcripts in *B. alternatus *was lower than in other *Bothrops *ESTs. Junqueira-de-Azevedo *et al. *[122] showed that svVEGF from *B. insularis *venom was able to increase vascular permeability and suggested that this protein could be involved in local and systemic vascular responses to envenoming.

#### Hyaluronidase

Hyaluronidase, which has an important role in facilitating venom diffusion from the site of inoculation through its degradation of hyaluronic acid in the extracellular matrix, is widespread in *Bothrops *venoms [123]. However, hyaluronidase has not generally been detected in transcriptomic and proteomic analyses of this genus, perhaps because of its low level of expression in venom glands. In agreement with this, we found only one EST similar to truncated hyaluronidase from *Bitis arietans *in the *B. alternatus *cDNA library.

#### L-Amino acid oxidase

L-Amino acid oxidase (LAO), which is widespread in *Bothrops *venoms [124], exerts a variety of biological activities, including interference with platelet aggregation, cytotoxicity and microbicidal activity [87,88]. These deleterious effects are mediated largely via the production of H_2_O_2 _during the oxidation of α-keto amino acids. LAO accounts for 0.5-2.6% of toxin transcripts in *Bothrops *venom gland transcriptomes [13-16] and has been purified and characterized from these venoms [88]. In the case of *B. alternatus*, we detected seven ESTs for this toxin in the nucleotide region: four of these were related to LAO from *B. jararaca *and three were ESTs in the 3'UTR related to *Ophiophagus hannah *LAO. In contrast to this low transcript abundance, LAO accounted for 6.9% of the venom proteins identified in a proteomic analysis of *B. alternatus *venom [90]. LAO purified from *B. alternatus *venom is an acidic (pI ~5.37), homodimeric (123 kDa) glycoprotein that induces platelet aggregation, causes edema, is bactericidal and slightly hemorrhagic [59]; this enzyme may contribute to the cytotoxicity of *B. alternatus *venom in cultured Madin-Darby canine kidney (MDCK) cells [125].

#### Three-finger toxins (3-FTx)

Three-finger toxins (3-FTx) consist predominantly of elapid neurotoxins, including α-neurotoxins, cardiotoxins and fasciculins, and a variety of less well-characterized venom proteins [126,127]. Originally thought to be restricted to elapids, 3-FTx have since been identified in crotalid [18,128] and colubrid [127,129,130] venom glands. 3-FTx exert a variety of biological activities that include blockade of acetylcholine (nicotinic and muscarinic) receptors, β-adrenergic receptors, L-type calcium channels and integrins, inhibition of acetylcholinesterase, and cardiotoxicity mediated by interation with phospholipids.

We identified 30 ESTs for 3-FTx in the *B. alternatus *venom gland library (0.56% of total ESTs; 2.4% of toxin ESTs) that shared 90% similarity with the intron II region of the gene for 3FTx-3 from *Sistrurus catenatus edwardsi *[128], but no hits with colubrid, elapid or *L. muta *[18] 3-FTx. Pahari *et al. *[128] also noted that the 3-FTx nucleotide and protein sequences of *S. c. edwardsi *bore no relationship to those of *L. muta*, a finding confirmed by phylogenetic analysis that placed the *L. muta *toxins distant from those of *S. c. edwardsi*. Complete sequencing of the *B. alternatus *genes would be helpful in determining the precise relationship between these 3-FTx and those of *S. c. edwardsi*.

This is the first identification of 3-FTx genes in *Bothrops *and, together with other studies, suggests that this class of toxins may occur in a variety of New World pitvipers, i.e., *Bothrops *[this study], *Sistrurus *[128] and *Lachesis *[18] (based on transcriptomic analyses) and *Atropoides mexicanus (nummifer) *(but not in *Atropoides picadoi*) (based on proteomic analysis) [131]. However, transcriptomic analyses have not detected these toxins in other New World pitvipers, e.g., *Agkistrodon piscivorus leucostoma *[132] and *C. d. collilineatus *[17], or in Old World pitvipers (*Agkistrodon *(*Deinagkistrodon*) *acutus *[133,134]) and vipers (*Bitis gabonica *[61] and *Echis *species [12,135]). In addition to this inter-generic variation, there is also intra-generic variation in the occurrence of 3-FTx. Thus, whereas 3-FTx have been detected in a transcriptomic analysis of *S. c. edwardsi *[128], these toxins have not been detected in a proteomic analysis of venoms from several members of this genus [136]. Likewise, *A. mexicanus (nummifer) *venom, but not that of *A. picadoi*, contains 3-FTx [128]. Such variation may reflect the low abundance of transcripts and proteins (making their detection difficult) and/or the non-uniform recruitment of these toxins into the venom proteome [128]. This could explain why transcriptomic [11,13-16] and proteomic [6,7,11,96,110,112,113,137] analyses have generally not detected these genes and proteins in *Bothrops *species.

The physiological relevance of 3-FTx to envenoming by *Bothrops *species is unclear, particularly in view of the low transcript abundance indicated by our results. However, *Bothrops *venoms cause neuromuscular blockade in avian and mammalian nerve-muscle preparations *in vitro *[138]. Although most of this activity has been attributed to the action of basic myotoxic PLA_2 _[91], 3-FTx could also contribute to this response through their ability to interfere with neuromuscular transmission.

#### Ohanin

Envenoming by *Bothrops *species results in local pain at the bite site [21,28]. Venom components implicated in this phenomenon include PLA_2_, metalloproteinases and serine proteinases that act via the release of endogenous mediators such as arachidonic acid metabolites (leucotrienes and prostaglandins), bradykinin, proinflammatory cytokines and neuronal nitric oxide [23,24,26]. In contrast, little is known of the involvement of other *Bothrops *venom proteins in this pain. The identification here of 24 ESTs with hits in the 3'UTR of the precursor for ohanin, an ~12 kDa hypolocomotion and hyperalgesic protein initially identified in the venom of the king cobra, *O. hannah*, and part of the vespryn family of proteins [139,140], indicates that other venom proteins, in addition to those indicated above, may be involved in *Bothrops *venom-induced pain. Since its initial identification, ohanin (vespryn) has been found in Australian elapids [9,141] and pitvipers such as *A. acutus *[133], *C. d. collilineatus *[17] and *L. muta *[18]. Our finding is the first report for ohanin in *Bothrops *venom glands. Together, these reports indicate that ohanins (and vespryns in general) are likely to be widespread in snake venoms where they may contribute to venom-induced hyperalgesia.

#### Taicatoxin-like protein

We detected two ESTs coding for a protein related to the serine proteinase inhibitor component of taicatoxin (TCX), a multimeric protein composed of an α-neurotoxin-like peptide (8 kDa), a neurotoxin PLA_2 _(16 kDa) and a serine proteinase inhibitor (7 kDa) (1:1:4 stoichiometry) initially isolated from the venom of the Australian taipan (*Oxyuranus scutellatus scutellatus*) [142,143]. TCX blocks high threshold, voltage-dependent calcium channel currents in cardiac membranes and cultured ventricular myocytes, although there may also be a role for the PLA_2 _activity in the action of this toxin [143,144]. The role of putative TCX-like proteins in *B. alternatus *is unclear but could contribute to the cardiovascular actions of this venom.

#### Other toxins

In addition to the foregoing toxins, we identified a low number of hits for several other toxins with BLAST results only in the nucleotide database. These hits included toxins related to cardiotoxins (12 reads), a muscarinic-like toxin (three reads) related to that of *Bungarus multicinctus*, precursors similar to post-synaptic α-neurotoxin NTX-2 from *Naja sputatrix *(one contig) and neurotoxin 6, and a molecule related to cobra-venom factor (one read).

### Putative toxins

Snake venoms contain a variety of nucleotidases (phosphodiesterases, 5'-nucleotidase, acid and alkaline phosphatases and ADP/ATPases) and nucleases (deoxyribonuclease - DNase and ribonuclease - RNase) that have a potentially important role in envenoming, particularly in affecting platelet aggregation and cardiovascular responses (hypotension, vascular permeability) [119,145]. In agreement with this, we obtained transcripts coding for a variety of genes related to some of these proteins, including an acidic DNase similar to a mouse DNAse IIα (lysosomal DNase) precursor (3 ESTs), ecto-5'-nucleotidases (one EST coding for the enzyme in zebrafish, *Danio rerio*, and another transcript coding for the same enzyme in horse, *Equus caballus*), ectonucleotide pyrophosphatase/phosphodiesterase 3 (two ESTs, one each for monkey, *Macaca mulatta*, and mouse, *Mus musculus*) and an adenosine deaminase related to *Xenopus laevis *enzyme (one transcript). Although several of these genes have been detected in other transcriptomic analyses [13,18,61,133,135], it is currently unclear whether the corresponding proteins are secreted into the venom or simply part of normal intracellular metabolism in the venom gland. We have purified phosphodiesterase [60], 5'-nucleotidase and an acidic DNase (DNase II) (unpublished findings) from *B. alternatus *venom, but since the structure of these proteins is unknown it is unclear to what extent they share similarity with the proteins coded by the genes identified in the venom gland ESTs. Ogawa *et al. *[146] have recently shown that *G. blomhoffi *ecto-5'-nucleotidase is structurally similar to this enzyme from various other vertebrates.

Other genes identified that could potentially be involved in envenoming included a cytokine-like protein (one EST similar to horse cytokine) (also identified in other snake venom ESTs [61,133]) and tumor necrosis factor (two ESTs similar to chicken, *Gallus gallus*, protein), both of which could contribute to the venom-induced local inflammatory response.

### Selected features of *B. alternatus *venom gland ESTs

#### Single nucleotide polymorphisms and simple repeats (microsatellites)

Polymorphisms in the nucleic acid sequences of snake venom proteins, particularly PLA_2_, have been identified in several species [147,148]. These genetic modifications arise from rapid gene duplication followed by single nucleotide polymorphisms (SNPs), with an increase in non-synonymous nucleotide substitutions that alter the DNA sequence encoding the protein. Ohno *et al. *[147] suggested that such alterations result in the rapid appearance of novel toxins with different biological activities. Our analysis revealed 132 putative SNPs in the *B. alternatus *transcriptome, of which 31 (16 non-synonymous and 15 synonymous substitutions) were located in ORFs, as determined based on alignment against the UniProt database. In addition, we identified 32 insertion-deletion polymorphisms (indels) (Table 1; Additional file 1). Although not extensively studied, detailed analysis of venom protein SNPs could be useful for population genetic studies and for assessing the importance of rapid sequence changes in generating the observed diversity of genes involved in venom production [148].

**Table 1:** Single nucleotide polymorphism (SNP) positions in the *B. alternatus *venom gland cDNA library.

Contig	Length (bp)	SNPs	First hit
Contig531	1006	9	Jararhagin [*Bothrops jararaca*]
Contig473	749	6	Zinc metalloproteinase-disintegrin ACLD precursor [*Gloydius brevicaudus*]
Contig425	844	6	Zinc metalloproteinase/disintegrin precursor [*Bothrops insularis*]
Contig500	715	5	Protein disulfide isomerase 3 precursor [*Gallus gallus*]
Contig322	700	4	ATP synthase F0 subunit 6 [*Agkistrodon piscivorus*]
Contig662	1213	3	Hypothetical 18K protein - goldfish mitochondrion
Contig310	568	2	Predicted: hypothetical protein [*Gallus gallus*]
Contig 54	758	2	Zinc metalloprotease-disintegrin halysase precursor [*Gloydius halys*]
Contig538	784	2	Ribosomal protein S2 [*Homo sapiens*]
Contig117	281	1	Similar to ribosomal protein L34 [*Monodelphis domestica*]
Contig285	594	1	Phospholipase A_2 _BITP01A precursor [*Bothrops insularis*]
Contig278	762	1	Unnamed protein product [*Homo sapiens*]
Contig181	763	1	Predicted: similar to calmodulin [*Mus musculus*]
Contig384	789	1	Piscivorin precursor [*Agkistrodon piscivorus*]
Contig 26	1001	1	Zinc metalloproteinase/disintegrin precursor [*Bothrops jararaca*]
Contig349	1051	1	Calreticulin [*Gallus gallus*]
Contig149	1313	1	Predicted: similar to PLCα, partial [*Ornithorhynchus anatinus*]
Contig335	1448	1	Cytochrome oxidase subunit 1 [*Campephilus guatemalensis*]
Contig840	1891	1	Zinc metalloproteinase-disintegrin jararhagin precursor [*Bothrops jararaca*]
Contig255	745	1	Predicted: similar to U2 (RNU2) small nuclear RNA [*Pan troglodytes*]

The polymorphisms were detected using the program TrueSNP, with the highest number being detected in genes related to jararhagin from *B. jararaca*.

The screening of unisequences with the tandem repeats finder tool resulted in the identification of 321 sequences with possible microsatellite regions; when annotated sequences with transposable elements (TEs) were excluded, 244 sequences with microsatellite regions were identified (data not shown). These sequences could be potentially useful for the development of molecular markers for characterization of the genetic variability and population structure of *B. alternatus *throughout its geographic distribution.

#### Transposable elements

Eukaryotic genomes contain a large number of repeated sequences, a high proportion of which may consist of transposable elements (TEs). In snakes, TEs have been previously identified in PLA_2 _genes from the venom gland of *Vipera ammodytes *[149,150]. These TEs are ruminant retroposons corresponding to 5'-truncated Bov-B long interspersed repeated DNA (LINE) and were identified in ammodytin L (a natural mutant of a group II PLA_2_) and ammodytoxin C (similar structure to other mammalian group II PLA_2_) genes. Alignments meeting our criteria (see Methods) allowed the identification of repeated elements in 492 unisequences, which corresponded to 9% of the *B. alternatus *ESTs (Additional file 2); (retro)transposons have also been identified in *B. insularis *[13] and *L. muta *[18]. Several TE families were identified among the *B. alternatus *transcripts, the most abundant being BovB, which was identified in 120 unisequences (Table 2). The expression of these TE families indicated that the genome harbored a large number of potentially active elements that gave rise to a variety of ESTs.

**Table 2:** Occurrence of transposable elements (TEs) in the *B. alternatus *venom gland cDNA library.

Family	Name	Genus in which originally described	*B. alternatus *unisequences
RTE	BovB	*Vipera *(snake)	120
CR1	CR1_HS	*Homo *(human)	19
L2	LINE2_CH1	*Crotalus *(snake)	15
hAT	HAT3_MD	*Monodelphis *(rat)	13
L2	LINE2_NT1	*Natrix *(snake)	8
hAT-Charlie	SPIN_Ml	*Myotis *(bat)	6
hAT-Charlie	URR1_Xt	*Xenopus *(frog)	4
L2	LINE2_WA1	*Walterinnesia *(snake)	3
hAT-Charlie	SPIN_Og	*Otolemur *(primate)	3
TcMar-Tc1	Tc1-3_Xt	*Xenopus *(frog)	3
hAT-Charlie	nhAT4b_ML	*Myotis *(bat)	2
hAT-Charlie	SPIN_NA_6_Et	*Echinops *(plant)	2
Sauria	AFESINE	*Azemiops *(snake)	1
R4	Rex6	*Takifugu *(fish)	1
TcMar-Tc1	Tc1-2_FR	*Takifugu *(fish)	1
TcMar-Tc1	TZF28B	*Danio *(fish)	1

The most frequently encountered TE was BovB, also identified in *Vipera*.

Further examination of the 492 unisequences harboring TEs revealed that 193 of them were fused to a sequence not included in the repeats database. To determine the identity of these sequences, we masked the TEs and BLASTed the masked unisequences against the GeneBank nr database. This approach revealed that in 80 unisequences, the TE was fused to a protein-coding gene (Additional file 3). Most transposition events involving protein-coding regions are deleterious to host genomes because they disrupt mRNA translation, localization and stability. However, since TEs associated with PLA_2 _genes have been found to be transcribed [136,137], we hypothesize that the TEs in *B. alternatus *may act as a source of genetic novelty by playing an important role in the origin and diversity of toxin genes.

#### Inverted repeats

**Figure 10:**
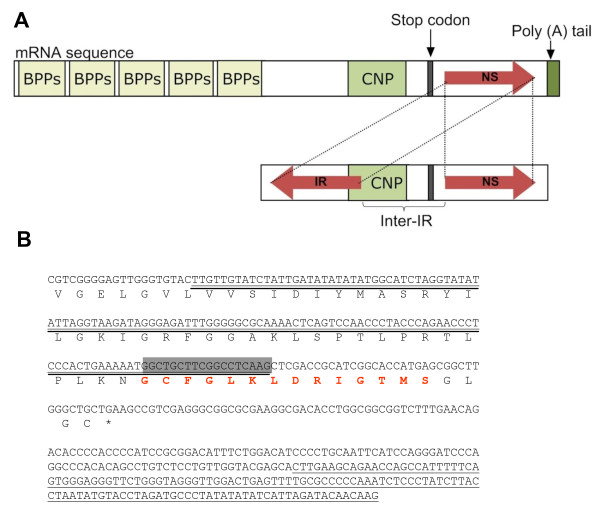
**An example of an inverted repeat in *B. alternatus *genes**. A) Structure of inverted repeat (IR) transcripts in a bradykinin-potentiating peptide/C-type natriuretic peptide (BPP/CNP) precursor gene identified in Contig649. The normal gene organization is shown in the upper part of (A), with a normal sequence (NS) of 132 nucleotides located in the 3'-UTR (red arrow, right) between the stop codon and the poly(A) tail. The lower part of (A) shows the location of the inverted repeat (IR; red arrow, left) relative to the NS. The IR occurred as a perfect repeat (palindrome) involving a portion of the CNP coding region. These two sequences were separated from each other by a 183-nucleotide segment referred to as the inter-IR domain. B) Nucleotide and protein sequences of Contig649 (466 bp long) for which 56 transcripts were obtained. The NS is identified by a single underline and the IR by a double underline. The gray box shows the CNP region involved in the IR and the CNP amino acid residues are indicated in red. * = stop codon.

The alignment of *B. alternatus *unisequences to corresponding reference genes indicated that some of the genes contained long inverted repeats (IR) in which a segment of the sequence was inverted from its original orientation in the reference gene (Additional file 4). An interesting example of such inversions involved genes for BPPs/CNPs, for which 16 unisequences with inverted repeats were observed (data not shown). Figure 10 shows the case in which an IR occurred in a portion of mRNA coding for part of a CNP. As mentioned above in the discussion of CNP genes, although EST studies have indicated the presence of genes for these peptides in *Bothrops *and *Lachesis *species, proteomic studies have not always been successful in isolating and identifying these peptides [11,111]. The reason for this discrepancy between EST and proteomic findings has not been adequately addressed but one explanation could be related to the occurrence of IR in these genes that may interfere with adequate gene transcription and/or translation *in vivo*, thereby preventing toxin production.

The origin of such IR transcripts is unclear. Theoretically, they could be artifacts generated by end-to-end joining of non-contiguous cDNA sequences and template switching by reverse transcriptase during cDNA synthesis; template switching can artificially delete portions of cDNAs and be wrongly interpreted as alternative transcripts. During first strand cDNA synthesis, poly(A)+ RNAs are primed by an oligo(dT) and when the reverse transcriptase reaches the 5'-end of the junction site in the mRNA, the nascent cDNA switches the template to the IR in the antisense mRNA that will now be used as a new template. However, since the homologous recombination between two RNA templates promoted by reverse transcriptase requires RNase H activity, and since the PowerScript™ Reverse Transcriptase used here for cDNA synthesis lacked RNase H activity, it seems unlikely that homology-dependent template switching could have generated these hybrid cDNAs artificially.

Further reasons that the IR observed here are unlikely to be artifacts include the following: 1) end-to-end joining during library construction would randomly ligate molecules and one would expect not only intra-molecular hybrids but also ligation of independently transcribed mRNAs, 2) in the case of an artifact, the transcript of highest abundance would be expected to be frequently ligated to other transcripts to form inter-molecular hybrids, but this was not observed in our library, and 3) the finding that the same ESTs containing inversions were present in multiple sequence reads from the same library and in independent libraries. Together, these observations suggest that this phenomenon was not an artifact.

### Comparison of the *B. alternatus *transcriptome with other *Bothrops *species

Following the initial report of gene expression analysis in the venom gland of *B. insularis *([13], recently updated by Valente *et al. *[11]), similar studies were reported for other *Bothrops *species, including *B. atrox *[16], *B. jararaca *[15] and *B. jararacussu *[14]. The availability of data for several *Bothrops *species provides an opportunity to compare related species and draw some general conclusions regarding venom composition in this genus. Few such transcriptomic comparisons are available for other genera [135].

To date, our study represents the largest EST database for *Bothrops*; the 5,350 ESTs reported here considerably exceed those reported for *B. atrox *(610) [16], *B. insularis *(initially 610, but updated to 2042) [11,13], *B. jararaca *(2318) [15] and *B. jararacussu *(549) [14]. We chose to generate a larger database than those previously reported for *Bothrops *species in an attempt to identify novel genes in addition to the main toxin groups already known for this genus. Indeed, this larger database allowed the detection of genes for toxins such as ohanin and 3-FTx, previously identified in other snake genera but not in *Bothrops*, in addition to non-venom proteins such as *DUSP6 *and thioredoxin.

The generation of a large database for *B. alternatus *did not significantly alter the profile of the most common toxin groups (metalloproteinases, BPPs/CNPs, PLA_2_, serine proteinases, C-type lectins, growth factors, etc.) when compared with other *Bothrops *species. This finding suggests that a large database is not essential for identification of the major toxin groups but can be useful for increasing the chances of detecting toxins with very low transcript abundance. This conclusion agrees with data for *B. insularis *in which increasing the EST database from 610 [13] to 2042 [11] ESTs did not markedly alter the relative proportions of metalloproteinases (41.7% vs. 43.2%, for 610 vs. 2042 ESTs), BPPs/CNPs (19.7% vs. 15.8%), C-type lectins (14.6% vs. 14.2%), serine proteinases (9.6% vs. 11.2%), PLA_2 _(6.7% vs. 5.4%), svVEGF (4.3% vs. 4.7%), LAO (2.6% vs. 3.5%), CRISPs (0.6% vs. 1.5%) and NGF (0.3% vs. 0.4%). In the present study, 78% of the hits corresponded to toxin transcripts, which compares favorably with values for other *Bothrops *species (54% - 78%) [11,13-16] and indicates that the relative proportion of toxin transcripts is not directly related to the size of the cDNA library.

Conversely, the generation of a large database resulted in a considerably greater number of no-hits: 70% in this study compared to 13% - 25% for other *Bothrops *studies [11,13-16] and 7% - 54% for other snake genera [12,18-20,61,128,132-135]. These no-hits reflect the limited amount of information available for *Bothrops *species and other snakes in venom gland EST databases, and represent a potentially rich source for the identification of novel toxins.

**Figure 11:**
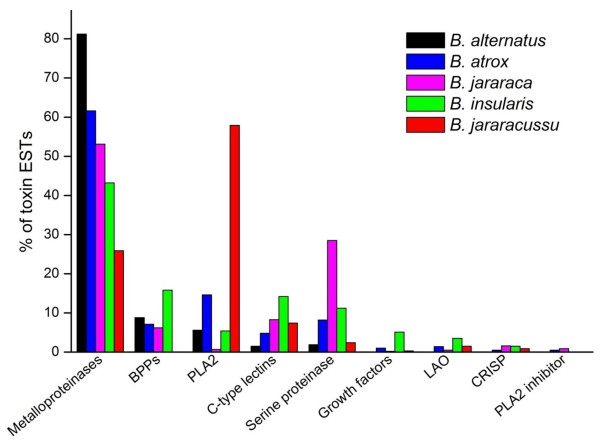
**Relative abundance of the major toxin classes in *Bothrops *venom glands determined by transcriptomic analysis**. Abundance is expressed as a percentage of the total toxin transcripts and was calculated by dividing the number of ESTs for each toxin family by the total number of toxin ESTs reported in each study. This standardization allowed direct comparisons across studies. Data sources other than *B. alternatus *were: *B. atrox *[16], *B. insularis *[11], *B. jararaca *[15] and *B. jararacussu *[14].

**Figure 12:**
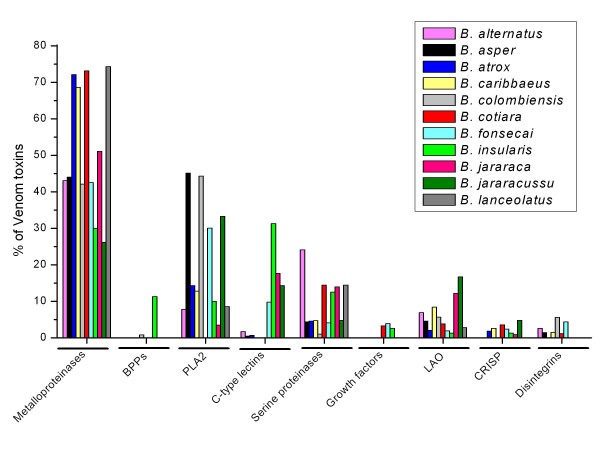
**Relative abundance of the major toxin classes in *Bothrops *venoms determined by proteomic analysis**. Abundance is expressed as a percentage of the total number of toxins identified in each analysis. Data sources were: *B. alternatus *[90], *B. asper *(Pacific population) [112,113], *B. atrox *(Brazilian population) [110], *B. caribbaeus *[7], *B. colombiensis *[6], *B. cotiara *[96], *B. fonsecai *[96], *B. insularis *[11], *B. jararaca *[103], *B. jararacussu *[97] and *B. lanceolatus *[7].

Figure 11 compares the relative abundance of the major toxin classes observed in *Bothrops *species based on EST analyses. In all cases, there was a predominance of metalloproteinases, BPPs, PLA_2_, serine proteinases and C-type lectins, with less abundant groups being LAO, CRISPs and growth factors (principally svVEGF and NGF). There was considerable inter-specific variation in the content of the major toxin. Thus, *B. alternatus *had the highest proportion of metalloproteinase transcripts among the five species, being more than three-fold more abundant than in *B. jararacussu*. PLA_2 _abundance was similar to *B. insularis*, greater than *B. jararaca *but less than *B. atrox *and *B. jararacussu*; the latter species was the only one in which PLA_2 _transcripts were more abundant than metalloproteinases (at least two-fold greater). The proportion of BPP/CNP transcripts in *B. alternatus *was similar to *B. atrox *and *B. jararaca *but about half that of *B. insularis*, while serine proteinases and C-type lectins were generally less abundant than in other *Bothrops *species. As indicated above, a lower content of serine proteinases and C-type lectins in the venom could account for the less severe coagulopathy observed clinically for envenoming by *B. alternatus *compared to other *Bothrops *species [36]. Despite the inter-specific variation in the relative proportion of toxin classes, these findings confirm that most *Bothrops *venom components can be classified into a few major groups. This conclusion agrees with proteomic analyses of *Bothrops *venoms that have also identified these groups as the major toxin families [6,7,11,90,96,97,110,112,113,137] (Figure 12). In addition to inter-specific variation, these proteomic studies have also reported individual, age-dependent and geographic variation in the toxin content of these major classes [110,112,137].

For five *Bothrops *species (*B. alternatus*, *B. atrox*, *B. insularis*, *B. jararaca *and *B. jararacussu*) there are transcriptomic and proteomic analyses that allow comparison of the toxin frequencies in the different classes. For metalloproteinases and PLA_2_, there is reasonably good agreement between the proportion of transcripts and the corresponding levels of these proteins detected in the venoms, whereas for other classes, e.g., BPPs, C-type lectins and serine proteinases, there are often marked discrepancies between the transcriptomic and proteomic data (cf. Figures 11 and 12). In the case of *B. alternatus*, there was good agreement between the proportion of ESTs (this study) and venom content [90] of PLA_2 _(5.5% vs 7.8%, respectively) and C-type lectins (1.4% vs. 1.7%), but considerable divergence between these two data sets in the case of metalloproteinases (81.4% vs. 43.1%), serine proteinases (1.9% vs. 24.1%) and LAO (0.6% vs. 6.9%) [this study and ref. [90]]. Divergent transcriptomic and proteomic results have also been observed for certain toxin groups in other snake genera, e.g., *Echis *species [12] and *L. muta *[10]. The causes and implications of such discrepancies have been discussed elsewhere [3] and indicate the need for caution in interpreting transcriptomic data as being representative of the final venom composition.

Finally, it should be noted that the principal toxin classes identified by transcriptomic and proteomic analyses are those that have been shown to contribute to the major local and systemic effects produced by these venoms, i.e., potent hemorrhagic activity (mediated by metalloproteinases), extensive myonecrosis (myotoxic PLA_2_), inflammatory responses (metallo- and serine proteinases, PLA_2_), coagulopathy (C-type lectins, serine proteinases, disintegrins) and cardiovascular actions (metalloproteinases, PLA_2_, serine proteinases such as kallikrein-like enzymes, and peptides such as BPPs) seen experimentally and clinically [21,22,26-30].

## Conclusions

An EST database containing 5,350 ESTs was produced for the venom gland of *B. alternatus*, the largest to date for the genus *Bothrops*. The venom gland expressed the major toxin groups (metalloproteinases, PLA_2_, serine proteases, C-type lectins and BPP/CNPs) identified in transcriptomic and proteomic analyses of other *Bothrops *species. In addition, genes for toxins (ohanin, 3-FTx and taicatoxin-like protein) and non-venom components (Dusp6 and thioredoxin) not previously detected in *Bothrops *venoms were also observed in this gland. The results described here expand our understanding of the composition of *B. alternatus *venom and help to explain the major symptoms associated with envenoming by this species.

## Abbreviations

BPP: bradykinin-potentiating peptide; CNP: C-type natriuretic peptide; CRISP: cysteine-rich secretory protein; DPP IV: dipeptidylpeptidase IV; EST: expressed sequence tags; 3-FTx: three-finger toxin; IR: inverted repeat; NGF: nerve growth factor; nr: non-redundant; nt: nucleotide database; ORF: open reading frame; SNP: single nucleotide polymorphism; SVMP: snake venom metalloproteinase; svVEGF: snake venom vascular endothelial growth factor; TE: transposable element; TLE: thrombin-like enzyme; UTR: untranscribed region.

## Competing interests

The authors declare that they have no competing interests.

## Authors' contributions

KCC was responsible for most of the experimental work, sequence annotation, data analysis, and wrote the paper under the supervision of SH. MJDS did part of the experimental work, provided day-to-day supervision of the experimental work and analyzed the results. GGLC performed the bioinformatics analyses, annotated the sequences and helped draft the manuscript. TTT helped with the bioinformatics analyses and manuscript preparation. LEVDB contributed to the sequence annotation. RV helped with the bioinformatics analyses. MM was involved in delineating the experimental design in the initial stages of the work and contributed to discussions of early experiments. SH conceived and wrote the project (together with KCC), supervised the project, helped in analyzing the results, and wrote the paper with KCC. All authors read and commented on the manuscript before submission.

## Supplementary Material

Additional file 1 **Table showing the positions and descriptions of SNPs identified in *B. alternatus *ESTs using the program QualitySNP **[72]. Click here for file

Additional file 2 **Table of transposable elements identified in *B. alternatus *ESTs**. Click here for file

Additional file 3 **Table of transposable elements fused with proteins in *B. alternatus *ESTs**. Click here for file

Additional file 4 **Table of inverted repeats (IR) identified in *B. alternatus *ESTs**. Click here for file
